# Dichotomy between T Cell and B Cell Tolerance to Neonatal Retroviral Infection Permits T Cell Therapy

**DOI:** 10.4049/jimmunol.1600734

**Published:** 2016-09-19

**Authors:** Bettina Mavrommatis, Lucie Baudino, Prisca Levy, Julia Merkenschlager, Urszula Eksmond, Tiziano Donnarumma, George Young, Jonathan Stoye, George Kassiotis

**Affiliations:** * Retroviral Immunology, The Francis Crick Institute, Mill Hill Laboratory, London NW7 1AA, United Kingdom;; † Retrovirus-Host Interactions, The Francis Crick Institute, Mill Hill Laboratory, London NW7 1AA, United Kingdom; and; ‡ Department of Medicine, Faculty of Medicine, Imperial College London, London SW7 2AZ, United Kingdom

## Abstract

Elucidation of the immune requirements for control or elimination of retroviral infection remains an important aim. We studied the induction of adaptive immunity to neonatal infection with a murine retrovirus, under conditions leading to immunological tolerance. We found that the absence of either maternal or offspring adaptive immunity permitted efficient vertical transmission of the retrovirus. Maternal immunodeficiency allowed the retrovirus to induce central Th cell tolerance in the infected offspring. In turn, this compromised the offspring’s ability to mount a protective Th cell–dependent B cell response. However, in contrast to T cells, offspring B cells were not centrally tolerized and retained their ability to respond to the infection when provided with T cell help. Thus, escape of retrovirus-specific B cells from deletional tolerance offers the opportunity to induce protective retroviral immunity by restoration of retrovirus-specific T cell help, suggesting similar T cell immunotherapies for persistent viral infections.

## Introduction

A cardinal feature of adaptive immunity is its acquired ability to discriminate self-antigens and nonself-antigens (1). Multiple layers of extrinsic regulation and intrinsic inhibition contribute to the prevention of autoimmune T cell and B cell responses (2–5). However, self–nonself discrimination, at the level of the somatically generated repertoire of TCRs and BCRs, respectively, is primarily achieved through clonal deletion of lymphocytes bearing strongly autoreactive receptors (2, 6). In contrast to other forms of self-tolerance, removal of such receptors from the TCR and BCR repertoire is irreversible.

In addition to lymphocytes reactive with germline-encoded self-antigens, clonal deletion can remove lymphocytes reactive with foreign Ags if they are introduced early in development (7, 8). Indeed, the phenomenon of acquired tolerance of foreign cells and Ags introduced into the developing organism was the basis for the landmark discovery of immunological tolerance (9). Similarly, the developmentally early introduction of maternal cells into the developing embryo naturally during gestation induces immunological tolerance to noninherited maternal Ags (10, 11). Depending on the degree of the resulting chimerism, deletional and regulatory mechanisms are thought to sustain tolerance of such Ags in the offspring (10, 11).

Mammalian offspring may acquire not only maternal cells during pregnancy; they may also acquire one or more infectious pathogens that can be transmitted vertically (12–14). These include maternal pathogens that are able to infect the offspring in utero (typically across the placenta), during birth (by contact with maternal blood or secretions), or after birth (usually via breast milk) and establish persistent infection (12–14). Important human viruses, such as rubella virus, several herpes viruses (CMV, HSV-1 and -2, and varicella-zoster virus), hepatitis viruses (hepatitis B virus and hepatitis C virus), enteroviruses (coxsackie virus and echovirus), and HIV-1 can be transmitted vertically, often with detrimental consequences (12–14). This mode of transmission is not restricted to viruses; mother-to-child transmission of bacterial and protozoan pathogens, such as *Mycobacterium tuberculosis* and *Plasmodium falciparum*, respectively, was documented (15, 16).

Many of these pathogenic infections have a direct impact on bone marrow and thymic function, thus impairing immune development (17). Additionally, by infecting these primary lymphoid organs, pathogens exploit clonal deletion of pathogen-specific lymphocytes to avoid or weaken immune recognition of their Ags (18). Clonal deletion of virus-specific thymocytes has long been shown in animal models of neonatal infection, including with murine leukemia virus (MLV) (19) and lymphocytic choriomeningitis virus (20). Moreover, HIV-1 infects the thymus and enhances MHC expression, which induces deletion of HIV-1–specific T cells (21), and in utero HIV-1 infection has a greater negative impact on HIV-1–specific T cell responses than infection during or after birth (22). Central tolerance induced by persistent pathogens is particularly efficient following neonatal infection, but it can also operate during infection of the adult host, as shown in mouse models for MLV (23) and *M. tuberculosis* infection (24).

Despite the considerable potential for persistent infection to induce central tolerance, thymic development of pathogen-specific T cells may proceed to some degree (18, 25). HIV-1–specific T cell responses can be detected in neonatally infected children, although these are often weak and functionally ineffective (22). Furthermore, CMV-specific T cells develop in CMV^+^ recipients of stem cell transplantation, indicating that thymic deletion can be avoided by at least some of the transplanted progenitors (26). The extent to which neonatal infection compromises the pathogen-specific TCR repertoire is not entirely known, but its manipulation could promote effective T cell responses during persistent infection. Furthermore, central tolerance caused by neonatal infection may follow different rules or operate to different degrees for T cells and B cells. Whether central B cell tolerance contributes to the impairment of the B cells’ response to persistent infection is unclear. Deeper understanding of the relationship between T cell and B cell tolerance and neonatal or chronic infection would also uncover potential causes for the ineffective B cell response that is often observed against such infections.

We studied the induction of virus-specific adaptive immune responses in a mouse model for neonatal infection with an MLV. The MLV that we used in this particular model is a recombinant between defective endogenous MLV proviruses, present in the C57BL/6 (B6) mouse germline (27). As a result, B6 mice are partially immunologically tolerant of its Ags (28). This recombinant MLV arose spontaneously and was transmitted efficiently in mice with B cell or Ab deficiencies but not in mice with T cell deficiencies (27). Although these studies highlighted the critical role of humoral, but not cellular, adaptive immunity in the control of vertical MLV transmission, the potential contribution of T cell help in the induction of the virus-specific Ab response was not clear. In this work, we show a dichotomy in T cell and B cell tolerance of neonatally acquired infection without the cover of maternal immunity, which further revealed that defective B cell responses were secondary to a primary defect in T cell help. Indeed, restoration of virus-specific Th cell immunity also restored virus-specific Ab responses in neonatally infected offspring, advocating the therapeutic potential of Th cells in persistent viral infection.

## Materials and Methods

### Mice

Inbred B6 and B6-backcrossed Rag1-deficient B6.129S7-*Rag1^tm1Mom^* (*Rag1*^−/−^) mice (29), MyD88-deficient B6.129P2-*Myd88^tm1Aki^* (*Myd88*^−/−^) mice (30), B6-congenic mice lacking *Emv2* (*Emv2*^−/−^) (28), TCRβ-transgenic EF4.1 mice with a higher frequency of ecotropic MLV (eMLV) env-specific CD4^+^ T cells (31), and TCRαβ doubly transgenic EVα3 mice with monoclonal eMLV env-specific CD4^+^ T cells (32) were described previously. EF4.1 mice were additionally crossed onto the *Emv2*^−/−^ background, and EVα3 mice were crossed onto the *Rag1*^−/−^*Emv2*^−/−^ background, to prevent T cell tolerance to *Emv2*-encoded Ags. Female *Emv2*^−/−^ mice were also crossed with male *Myd88*^−/−^ mice, and their progeny were intercrossed to create virus-free *Myd88*^−/−^*Emv2*^−/−^ mice. These strains were maintained in specific pathogen–free facilities at the Francis Crick Institute and used between 8 and 12 wk after birth. All animal experiments were approved by the ethical committees of The Francis Crick Institute, Mill Hill Laboratory and were conducted according to local guidelines and regulations.

### Retroviral infections

The *Rag1*^−/−^ mice used in this study carry an infectious eMLV, referred to as RARV2, which is naturally transmitted vertically (27). The *Myd88*^−/−^ mice used carry a similar infectious eMLV. Both of these viruses are the result of recombination between replication-defective endogenous MLVs (27). To achieve vertical transmission to wild-type (WT) progeny, virus-carrier female mice were crossed with the indicated males, and the progeny were analyzed for virus replication. Additionally, where indicated, 1-d-old pups born to virus-free dams were foster nursed by virus-carrier females. For the experiments described in Fig. 2, mice were additionally infected with Friend virus (FV), a retroviral complex between the Friend strain of MLV (F-MLV) and the replication-defective spleen focus-forming virus (33, 34). FV stocks were propagated in vivo and prepared as 10% w/v homogenate from the spleen of 12-d infected BALB/c mice, as previously described (35, 36). Mice received an inoculum of ∼1000 spleen focus-forming units of FV. Stocks were free of *Mycoplasm*a spp., Sendai virus, murine hepatitis virus, parvoviruses 1 and 2, reovirus 3, Theiler’s murine encephalomyelitis virus, murine rotavirus, ectromelia virus, murine CMV, K virus, polyomavirus, Hantaan virus, murine norovirus, lymphocytic choriomeningitis virus, murine adenoviruses FL and K87, and lactate dehydrogenase–elevating virus.

### Retrovirus expression and quantitation

Cellular expression of the RARV2/*Emv2* env open-reading frame was quantified by real-time quantitative RT-PCR, as previously described (27). Briefly, total spleen or liver RNA was reverse transcribed into cDNA with the High-Capacity Reverse Transcription Kit (Applied Biosystems, Carlsbad, CA) and used as template for the amplification of target gene transcripts. The following primers (Eurofins MWG Operon, Ebersberg, Germany), specific for the cDNA produced from the spliced RARV2/*Emv2 env* mRNA were used: forward: 5′-CCAGGGACCACCGACCCACCGT-3′ and reverse: 5′-TAGTCGGTCCCGGTAGGCCTCG-3′.

Values were normalized and plotted according to expression of *Hprt* in the same samples, which was detected with the following primers: forward: 5′- TGTATACCTAATCATTATGCCGAG-3′ and reverse: 5′-CATCTCGAGCAAGTCTTTCA-3′.

RARV2 plasma viremia was also quantified by real-time quantitative RT-PCR. Fixed volumes of plasma samples were used for the isolation of RNA, using the automated QIAcube workstation (QIAGEN, Hilden, Germany) and cDNA synthesis. RARV2 copies were calculated using the following primers specific for the cDNA produced from full-length RARV2/*Emv2* RNA: forward: 5′-AGGCTGTTCCAGAGATTGTG-3′ and reverse: 5′-TTCTGGACCACCACACGAC-3′.

Genomic B6 DNA, containing two copies of *Emv2*/diploid genome, was used as a standard to convert values into copies of genomic RNA per ml of plasma.

### Retrovirus neutralizing and infected cell-binding Ab assays

Titers of retrovirus-infected cell-binding Abs were determined by flow cytometry following primary staining of *Mus dunni* cells, infected with RARV2 or F-MLV, using serum samples and secondary staining with fluorescently labeled anti-mouse IgG1 (clone A85-1), anti-mouse IgG2a/c (clone R19-15), anti-mouse IgG2b (clone R12-3), or anti-mouse IgM (clone R6-60.2) Abs (all from BD), as previously described (28). Although the R19-15 mAb has higher affinity for IgG2a, it can be used effectively for detection of the B6-specific IgG2c isotype (28). The median fluorescence intensity obtained at 1:100 serum dilution and normalized by the median fluorescence intensity of control serum was taken as the arbitrary binding titer. Serum titers of RARV2- or F-MLV–neutralizing Abs were measured using a previously described method (37). Briefly, *M. dunni* cells (38) were transduced with the XG7 replication-defective retroviral vector, expressing GFP from a human CMV promoter and a neomycin-resistance gene under the control of the LTR (39). *M. dunni*–XG7 cells were then infected with RARV2 or with F-MLV, and supernatant, which contained the pseudotyped XG7 vector, was harvested. Serial dilutions of sera from infected mice were mixed with ∼1500 IU/ml pseudotyped XG7 vector and allowed to incubate for 30 min at 37°C in IMDM cell culture medium containing 5% FCS. Mixtures were added to untransduced *M. dunni* cells and incubated for 3 d. The percentage of GFP^+^ *M. dunni* cells at the end of the incubation period was assessed by flow cytometry, and the dilution of serum that resulted in 75% neutralization (i.e., 75% reduction in the percentage of GFP^+^ *M. dunni* cells) was taken as the neutralizing titer.

### Flow cytometry

Cell suspensions from spleens were stained with directly conjugated surface Ag–specific Abs, obtained from eBioscience (San Diego, CA), Caltag/Invitrogen, BD Biosciences (San Jose, CA), or BioLegend (San Diego, CA). MLV surface unit was detected using 83A25 mAb (40) (rat IgG2a, anti-MLV surface unit) as the primary reagent, followed by staining with a biotinylated anti-rat IgG2a Ab (clone RG7/1.30; BD Biosciences) as the secondary reagent and a streptavidin-PE Texas Red conjugate (CALTAG/Invitrogen) as the tertiary reagent. FV-infected cells were detected by flow cytometry using surface staining for the glycosylated product of the viral *gag* gene (glyco-Gag) using the matrix-specific mAb 34 (mouse IgG2b), followed by an anti-mouse IgG2b-FITC secondary reagent (BD Biosciences). Multicolor cytometry was performed on FACSCanto II, LSRFortessa X-20 (both from BD Biosciences), and CyAn (Dako, Fort Collins, CO) flow cytometers and analyzed with FlowJo v10 (TreeStar, Ashland, OR) or Summit v4.3 (Dako) software.

### Lymphocyte isolation and transfer

Single-cell suspensions were prepared from the spleens and lymph nodes of donor mice. EF4.1 *Emv2*^−/−^ CD45.2^+^ mice were donors of env-reactive CD4^+^ T cells. RARV2 neonatally infected B6 CD45.2^+^ and uninfected B6 CD45.1^+^CD45.2^+^ mice were donors of B cells for the experiments described in Fig. 6D and 6E. CD4^+^ T cells and B cells were isolated using CD4 and B220 Abs, respectively, and following immunomagnetic positive selection (STEMCELL Technologies, Vancouver, BC, Canada), at >96% purity. Isolated cells were injected into the indicated recipients via the tail vein.

### Statistical analyses

Statistical comparisons and regressions were performed using SigmaPlot 13.0 (Systat Software, Erkrath, Germany). Parametric comparisons of normally distributed values that satisfied the variance criteria were made using unpaired Student *t* tests or one-way ANOVA. Data that did not pass the variance test were compared using the nonparametric two-tailed Mann-Whitney rank-sum test or the ANOVA on ranks test.

## Results

### Maternal immunodeficiency permits efficient infection of immunocompetent progeny

**FIGURE 1.**
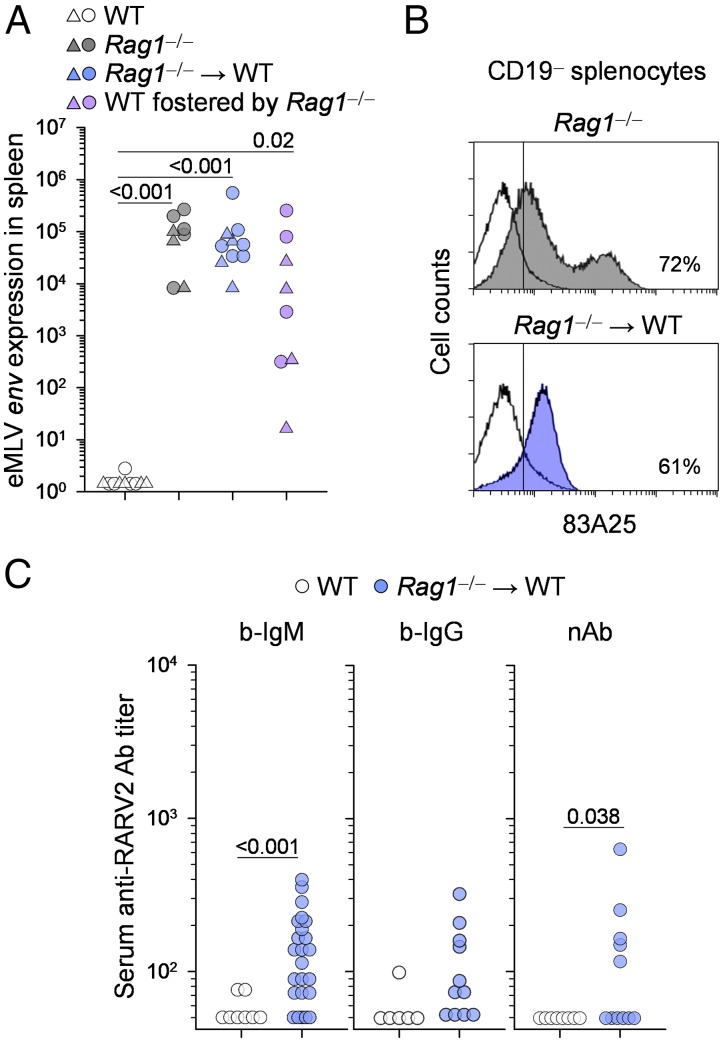
Efficient retrovirus transmission from immunodeficient dams to immunocompetent progeny. (**A**) RARV2/*Emv2* eMLV *env* expression, relative to *Hprt* expression, in the spleens of uninfected control mice (WT), virus-carrier immunodeficient mice (*Rag1*^−/−^), immunocompetent (*Rag1*^+/−^) mice born to virus-carrier *Rag1*^−/−^ dams (*Rag1*^−/−^ → WT), and WT pups foster nursed by virus-carrier *Rag1*^−/−^ dams (WT fostered by *Rag1*^−/−^). Each symbol is an individual mouse; different symbols denote litters of individual dams. The *p* values were calculated using the ANOVA on ranks test. (**B**) Flow cytometric detection of MLV gp70 expression, using the 83A25 Ab, in CD19^−^ splenocytes from virus-carrier *Rag1*^−/−^ mice or immunocompetent mice born to virus-carrier *Rag1*^−/−^ dams (*Rag1*^−/−^ → WT). Plots are representative of four mice per group. (**C**) Titers of RARV2-infected cell-binding IgM and IgG Abs and of RARV2-neutralizing Abs in the sera of uninfected WT mice and immunocompetent mice born to virus-carrier *Rag1*^−/−^ dams (*Rag1*^−/−^ → WT). Each symbol is an individual mouse. The *p* values were calculated using the Mann–Whitney rank-sum test.

To examine the contribution of maternal immunity to the prevention of vertical infection, we set up a model for retroviral transmission from virus-carrier T cell– and B cell–deficient *Rag1*^−/−^ mice to immunocompetent WT pups (*Rag1*^−/−^ → WT). *Rag1*^−/−^ female mice carrying an infectious eMLV (RARV2), which arose de novo through recombination between defective endogenous MLV proviruses (27), were crossed with WT male mice. Inheritance of a functional *Rag1* allele from the WT male restored immune competence in the progeny. Expression levels of eMLV *env* in the spleen, a readout for infection (27), were as high in all progeny as in the parental virus-carrier *Rag1*^−/−^ mice and were significantly higher than in uninfected WT mice (Fig. 1A). Similarly high expression of eMLV was noted in the spleens of WT pups that were foster nursed by virus-carrier *Rag1*^−/−^ dams (Fig. 1A), indicating high efficiency of neonatal virus transmission. Moreover, MLV env protein was expressed at higher levels on CD19^−^ splenocytes from progeny than from uninfected WT mice, although they were not as high as on splenocytes from virus-carrier *Rag1*^−/−^ mice (Fig. 1B).

Uninfected WT mice carry the precursors of RARV2 as defective endogenous MLV proviruses, the protein expression of which may elicit an MLV-specific Ab response with age (41). Nevertheless, uninfected WT mice did not develop detectable RARV-specific–infected cell-binding or virus-neutralizing Abs during these experiments (Fig. 1C). In contrast, many neonatally infected WT mice mounted an RARV2-specific Ab response (Fig. 1C), despite being uniformly unable to control retroviral infection (Fig. 1A). However, this Ab response primarily involved RARV2-infected cell-binding IgM Abs, whereas RARV2-infected cell-binding class-switched IgG Abs were not significantly induced, and RARV2-neutralizing Abs were weakly induced in a fraction of the mice (Fig. 1C). In individual mice, RARV2-neutralizing Ab titers correlated poorly with titers of RARV2-infected cell-binding IgM or IgG Abs (Supplemental Fig. 1), as would be expected given that many neonatally infected WT mice lacked RARV2-neutralizing Abs. Thus, neonatal infection of WT pups elicited partial and weak RARV2-neutralizing Ab responses.

### Neonatally infected WT mice mount a humoral response to superinfection

**FIGURE 2.**
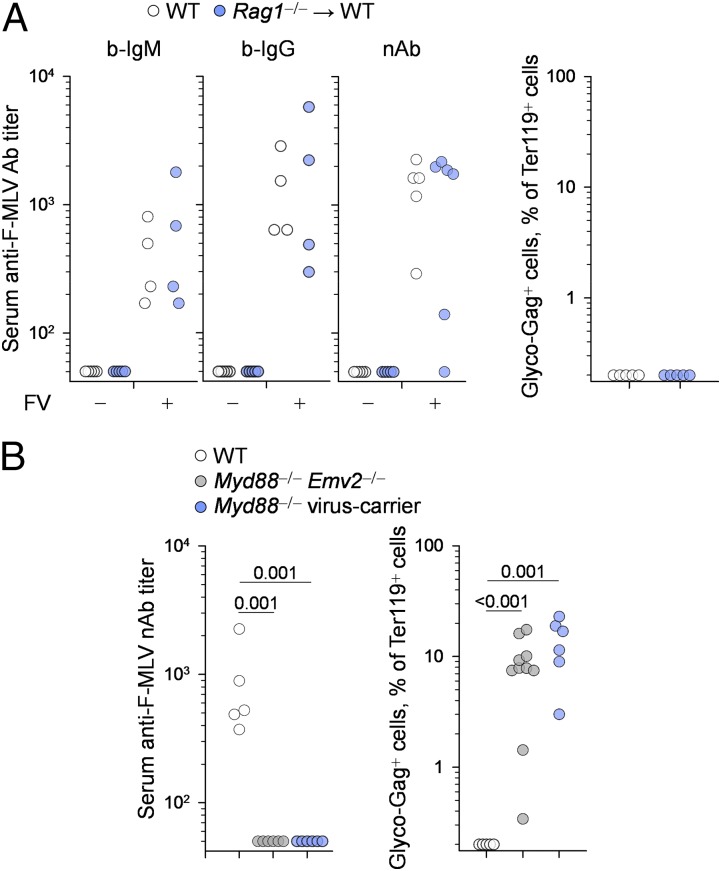
Neonatally infected WT mice resist superinfection with homologous virus. (**A**) Titers of F-MLV–infected cell-binding IgM and IgG Abs and of F-MLV–neutralizing Abs in the sera of WT mice not infected with RARV2 mice and RARV2 neonatally infected immunocompetent mice born to a virus-carrier *Rag1*^−/−^ dam (*Rag1*^−/−^ → WT), before (FV−) and 21 d after (FV+) FV infection (left panels). Frequency of FV-infected (glyco-Gag^+^) cells within Ter119^+^ cells in the spleens of the same mice 21 d after FV infection (right panel). Each symbol is an individual mouse from a single experiment. WT and *Rag1*^−/−^ → WT groups were not statistically different (Mann–Whitney rank-sum test). (**B**). Titers of F-MLV–neutralizing Abs in the sera of WT mice not infected with RARV2, virus-free *Myd88*^−/−^*Emv2*^−/−^ mice, and virus-carrier *Myd88*^−/−^ mice 21 d after FV infection (left panel). Frequency of FV-infected (glyco-Gag^+^) cells within Ter119^+^ cells in the spleens of the same mice (right panel). Each symbol is an individual mouse pooled from two independent experiments. The *p* values were calculated with the ANOVA on ranks test.

To investigate whether neonatal infection of WT mice caused general immune defects or simply immunological tolerance specifically to RARV2, we superinfected these mice with FV, a retroviral complex of replication-competent F-MLV and replication-defective spleen focus-forming virus. Importantly, the ecotropic envelope glycoproteins of F-MLV and RARV2 share 79% amino acid identity (27). Despite such homology, sera from WT mice neonatally infected with RARV2 exhibited no F-MLV–infected cell-binding or F-MLV–neutralizing activity prior to FV infection (Fig. 2A, left panels). In contrast, at 21 d post–FV infection, WT mice that also had been neonatally infected with RARV2 mounted infected cell-binding and virus-neutralizing Abs against F-MLV at titers that were comparable to those in WT mice not infected with RARV2 (Fig. 2A, left panels). Moreover, WT mice effectively controlled FV infection, irrespective of prior neonatal infection with RARV2 (Fig. 2A, right panel). Thus, neonatal RARV2 infection did not appear to compromise the ability of WT mice to mount an Ab response to, or control replication of, a homologous retrovirus.

Given the homology of their envelope glycoproteins, both of which use the mCAT-1 cellular receptor, it was possible that the resistance of RARV2-infected WT mice to FV superinfection was due to receptor interference (42). We first confirmed the potential of RARV2 to interfere with FV infection. To exclude indirect effects of adaptive immunity, we compared RARV2-carrier *Rag1*^−/−^ mice with RARV2-free *Rag1*^−/−^*Emv2*^−/−^ mice. Following FV infection, the frequency of FV-infected cells in Ter119^+^ erythroid precursor cells was significantly higher in RARV2-free *Rag1*^−/−^*Emv2*^−/−^ mice than in RARV2-carrier *Rag1*^−/−^ mice (Supplemental Fig. 2). As expected, expression of envelope proteins of MLVs other than F-MLV, detected with the 83A25 mAb, was found only in virus-carrier *Rag1*^−/−^ mice (Supplemental Fig. 2). Thus, the RARV2-carrier status significantly reduced susceptibility to FV infection, independently of adaptive immunity.

To compare the effect of receptor interference and Ab-mediated resistance to FV infection, we used virus-free *Myd88*^−/−^*Emv2*^−/−^ mice, in which the lack of any ecotropic envelope glycoprotein expression precludes receptor interference, and virus-carrier *Myd88*^−/−^ mice, in which receptor interference is as strong as in neonatally infected WT mice. Notably, the Ab response to FV would be defective in virus-free *Myd88*^−/−^*Emv2*^−/−^ mice and virus-carrier *Myd88*^−/−^ mice as a result of MyD88 deficiency, but receptor interference would only be present in the latter. Neither of these strains mounted a detectable F-MLV–neutralizing Ab response, whereas both were unable to control FV infection (Fig. 2B). These results indicated that resistance of RARV2 neonatally infected WT mice to FV infection is mediated by a MyD88-dependent Ab response to FV, rather than by receptor interference.

### Maternal and offspring immunity cooperate in the control of vertical transmission

**FIGURE 3.**
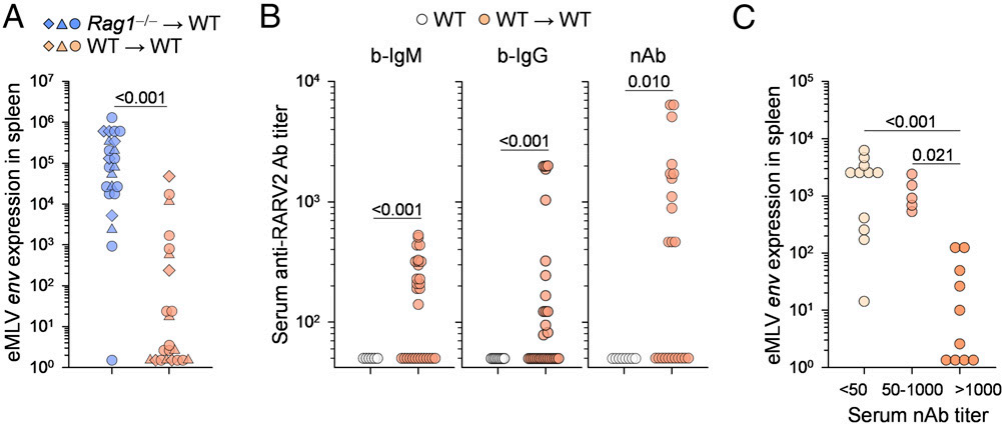
Inefficient vertical transmission in immunocompetent mice. (**A**) RARV2/*Emv2* eMLV *env* expression, relative to *Hprt* expression, in the spleens of immunocompetent (*Rag1*^+/−^) mice (F_1_) born to virus-carrier *Rag1*^−/−^ dams (*Rag1*^−/−^ → WT) and immunocompetent (*Rag1*^+/−^ or *Rag1*^+/+^) mice (N_2_) born to virus-carrier immunocompetent dams (WT → WT). Each symbol is an individual mouse; different symbols denote litters of individual dams. The *p* values were calculated by the Mann–Whitney rank-sum test. (**B**) Titers of RARV2-infected cell-binding IgM and IgG Abs and of RARV2-neutralizing Abs in the sera of uninfected WT mice and immunocompetent mice (N_2_) born to virus-carrier immunocompetent dams (WT → WT). Each symbol is an individual mouse pooled from three independent experiments. The *p* values were calculated by the Mann–Whitney rank-sum test. (**C**) Correlation between RARV2/*Emv2* eMLV *env* expression and serum titers of RARV2-neutralizing Abs in immunocompetent mice (N_2_) born to virus-carrier immunocompetent dams (WT → WT). Mice were grouped into Ab negative (titer < 50), intermediate (titer 50–1000), and high (titer > 1000). Symbols represent the individual mice described in (B). The *p* values were calculated by the ANOVA on ranks test.

Our findings suggested that vertical transmission of a retrovirus from an immunodeficient dam left the immunocompetent progeny unable to mount a protective humoral response. However, this experimental set-up did not include the contribution of maternal immunity. Therefore, we examined whether immunocompetent progeny would be better able to control vertical transmission from an immunocompetent dam. To this end, we crossed virus-carrier WT females (F_1_, born to virus-carrier *Rag1*^−/−^ mice) with WT males and assessed retrovirus transmission to the next generation (N_2_, WT → WT). Remarkably, the majority (68%) of N_2_ mice were virus-free (Fig. 3A) and collectively had significantly elevated titers of RARV2-infected cell-binding IgG or RARV2-neutralizing Abs (Fig. 3B). RARV2-neutralizing Ab titers correlated reasonably well with titers of RARV2-infected cell-binding IgG or IgM Abs, which also correlated between them (Supplemental Fig. 3). Importantly, resistance to neonatal infection strongly correlated with the development of high titers of RARV2-neutralizing Abs (>1000) in the progeny (Fig. 3C). Thus, vertical transmission of this retrovirus from immunocompetent dams to immunocompetent progeny was inefficient.

**FIGURE 4.**
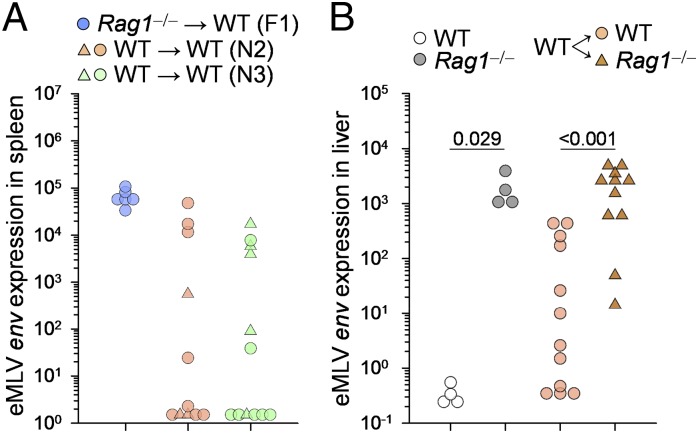
Cooperation of maternal and offspring immunity in the control of neonatal infection. (**A**) RARV2/*Emv2* eMLV *env* expression, relative to *Hprt* expression, in the spleens of immunocompetent (*Rag1*^+/−^) mice born to virus-carrier *Rag1*^−/−^ dams (*Rag1*^−/−^ → WT [F_1_]), immunocompetent (*Rag1*^+/−^ or *Rag1*^+/+^) mice born to virus-carrier immunocompetent F_1_ dams (WT → WT [N_2_]), and immunocompetent (*Rag1*^+/−^ or *Rag1*^+/+^) mice born to virus-carrier immunocompetent N_2_ dams (WT → WT [N_3_]). Each symbol is an individual mouse; different symbols denote litters of individual dams. The N_2_ and N_3_ groups were not statistically different (ANOVA on ranks test). (**B**) RARV2/*Emv2* eMLV *env* expression, relative to *Hprt* expression, in the livers of uninfected control mice (WT), virus-carrier immunodeficient mice (*Rag1*^−/−^), and immunocompetent (*Rag1*^+/−^or *Rag1*^+/+^) or immunodeficient (*Rag1*^−/−^) littermates born to virus-carrier immunocompetent F_1_ dams. Each symbol is an individual mouse. Two successive litters of two virus-carrier immunocompetent F_1_ dams were pooled. The *p* values were calculated by the Mann–Whitney rank-sum test (littermate comparison).

Next, we used two approaches to confirm that maternal immunity to RARV2 was not solely responsible for the inefficient transmission of this virus to immunocompetent progeny. First, virus-carrier N_2_ WT (*Rag1*^+/−^) females were crossed with WT males to obtain an additional generation (N_3_, WT → WT). Again, a proportion of the progeny expressed high levels of vertically transmitted virus (Fig. 4A), indicating that RARV2 can be transmitted, albeit inefficiently, through successive generations of WT mice. Second, virus-carrier N_2_ WT (*Rag1*^+/−^) females were crossed with *Rag1*^−/−^ males, producing immunodeficient (*Rag1*^−/−^) and immunocompetent (*Rag1*^+/−^) littermates. Expression of vertically transmitted RARV2 was measured in the livers, instead of the spleens, to control for the differences in the size and cellular composition of the latter organs between *Rag1*^−/−^ and *Rag1*^+/−^ mice. Notably, only immunocompetent littermates were able to control or clear RARV2 infection (Fig. 4B), demonstrating the requirement for offspring immunity, in addition to maternal immunity, in resistance to vertical infection.

### Maternal immunodeficiency causes T cell tolerance in neonatally infected offspring

**FIGURE 5.**
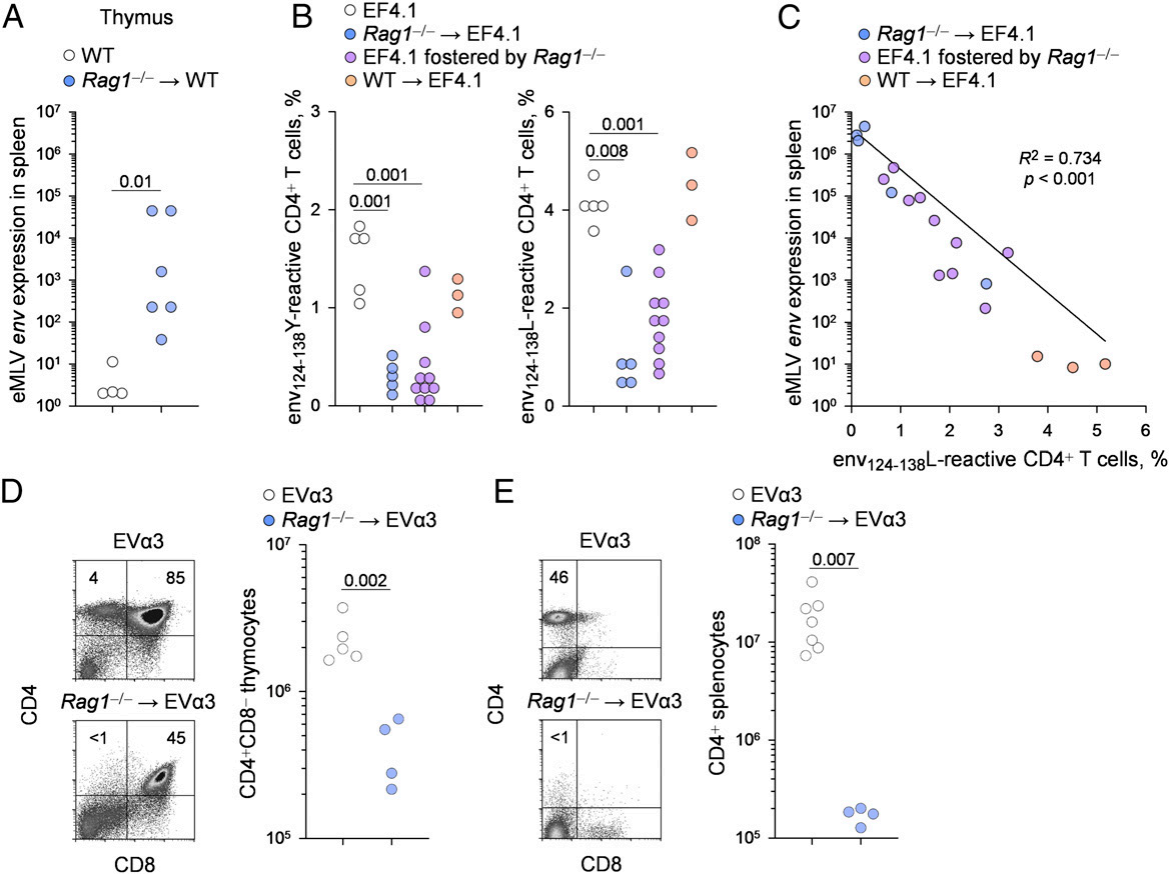
Maternal immunodeficiency causes T cell tolerance in neonatally infected offspring. (**A**) RARV2/*Emv2* eMLV *env* expression, relative to *Hprt* expression, in the spleens of uninfected control mice (WT) and immunocompetent (*Rag1*^+/−^) mice born to virus-carrier *Rag1*^−/−^ dams (*Rag1*^−/−^ → WT). Each symbol is an individual mouse. One of two experiments is shown. The *p* value was calculated by the Mann–Whitney rank-sum test. (**B**) Frequency of env_124–138_Y- and env_124–138_L-reactive cells in splenic CD4^+^ T cells from uninfected EF4.1 mice (EF4.1), EF4.1 mice born to virus-carrier *Rag1*^−/−^ dams (*Rag1*^−/−^ → EF4.1), EF4.1 pups foster nursed by virus-carrier *Rag1*^−/−^ dams (EF4.1 fostered by *Rag1*^−/−^), and EF4.1 mice born to virus-carrier immunocompetent dams (WT → EF4.1). Each symbol is an individual mouse pooled from two independent experiments. The *p* values were calculated by the ANOVA on ranks test. (**C**) Correlation between frequency of env_124–138_L-reactive cells in CD4^+^ T cells and RARV2/*Emv2* eMLV *env* expression, relative to *Hprt* expression, in the spleens of the same groups of mice as in (B). (**D**) Flow cytometric profile (left panels) and absolute number (right panel) of CD4^+^ mature thymocytes in uninfected EVα3 mice (EVα3) and EVα3 mice born to virus-carrier *Rag1*^−/−^ dams (*Rag1*^−/−^ → EVα3). (**E**) Flow cytometric profile (left panels) and absolute number (right panel) of splenic CD4^+^ T cells in the same mice as in (D). In (D) and (E), symbols represent individual mice from two separate litters. The *p* values were calculated by the Mann–Whitney rank-sum test.

Development of higher titers of retrovirus-specific class-switched or neutralizing Abs in the offspring of immunocompetent, but not immunodeficient, dams suggested differences in the availability of T cell help, which typically is required for the induction of such Abs. Thus, we investigated the effect of maternal immunity on the ability of the offspring to develop and deploy retrovirus-specific Th cells. Vertical transmission from virus-carrier immunodeficient *Rag1*^−/−^ mice led to efficient infection of the thymi of immunocompetent WT progeny (*Rag1*^−/−^ → WT) (Fig. 5A), which would affect thymic selection of RARV2-specific T cells. To quantify this effect, we used a TCRβ-transgenic strain (EF4.1) with an increased frequency of CD4^+^ T cells reactive with the dominant H2-A^b^–restricted epitope within the eMLV envelope glycoprotein (env_124–138_) (31). RARV2 and F-MLV envelopes differ in this region by a single amino acid residue (Y and L, respectively, at position 128) and, importantly, EF4.1 mice produce CD4^+^ T cell clones that react with either of these two variants (28).

In uninfected EF4.1 mice, the RARV2 precursor *Emv2* endogenous MLV causes partial deletion of env_124–138_Y-reactive CD4^+^ T cells (28). Nevertheless, EF4.1 mice that were neonatally infected with RARV2, either by being born to or foster nursed by virus-carrier *Rag1*^−/−^ dams, lost nearly all reactivity to the env_124–138_Y epitope in the periphery (Fig. 5B). In contrast, EF4.1 mice born to virus-carrier WT dams retained env_124–138_Y reactivity (Fig. 5B). Similar results were obtained when reactivity to the env_124–138_L epitope was tested (Fig. 5B). Moreover, the degree of residual CD4^+^ T cell reactivity to the latter epitope in these groups of mice exhibited a strong inverse correlation with levels of RARV2 replication (Fig. 5C). These data indicated that vertical transmission of RARV2 from immunodeficient dams compromised the ability of the progeny to mount an RARV2-specific CD4^+^ T cell response.

To confirm that lack of peripheral env_124–138_ reactivity in EF4.1 mice born to virus-carrier *Rag1*^−/−^ dams was due to central deletion of env_124–138_-reactive CD4^+^ T cells, rather than peripheral tolerance or unresponsiveness, we used a TCRαβ doubly transgenic strain (EVα3) that also reacts with both env_124–138_ variants (28, 32). In comparison with uninfected EVα3 mice, the mice neonatally infected with RARV2 by their virus-carrier *Rag1*^−/−^ mothers almost completely lacked CD4^+^ mature thymocytes (Fig. 5D) and CD4^+^ splenocytes (Fig. 5E). Thus, retroviruses vertically transmitted from immunodeficient dams prevented thymic development of retrovirus-specific CD4^+^ T cells.

### Altered, but not absent, retrovirus-specific B cell responses in vertically infected hosts

**FIGURE 6.**
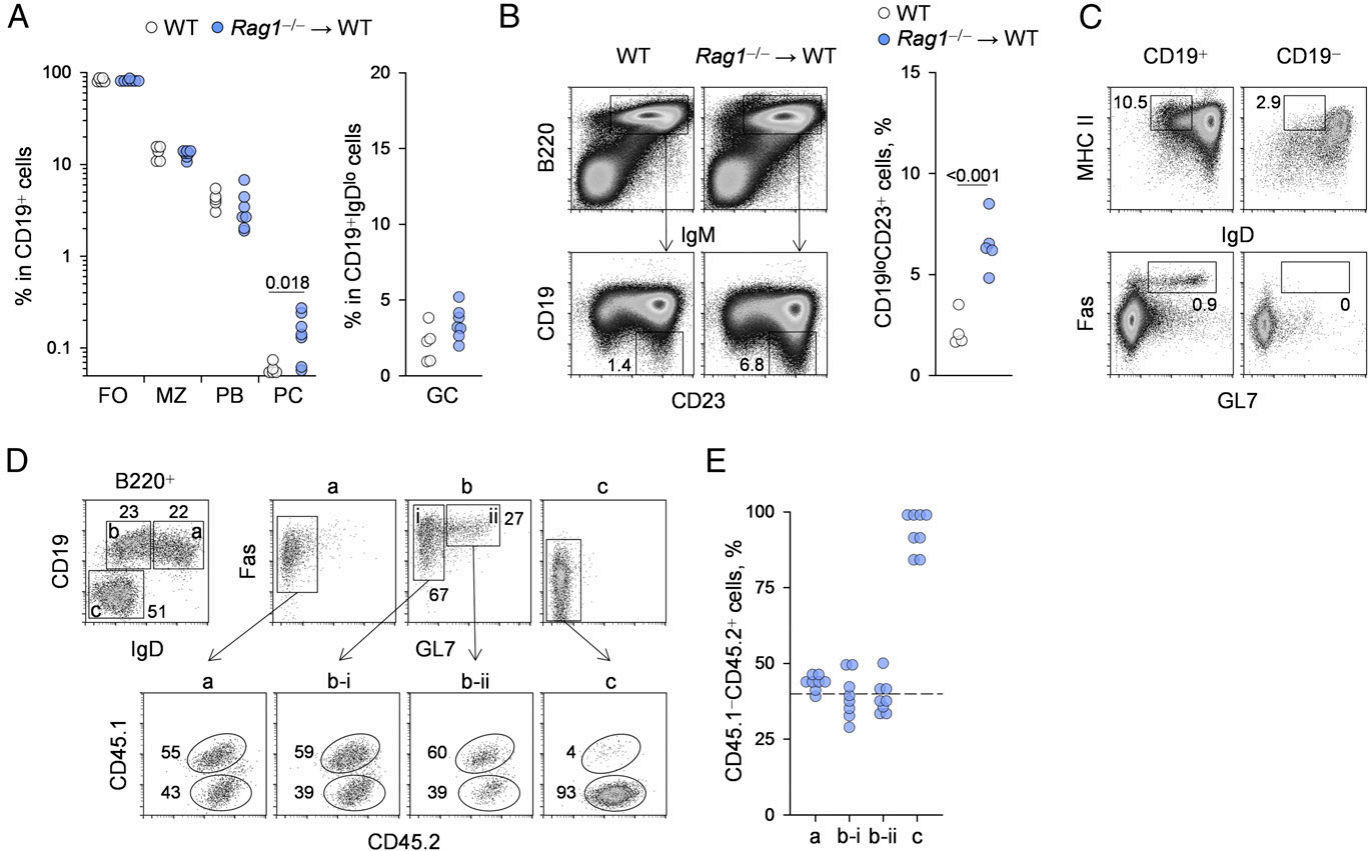
B cell responses in vertically infected hosts. (**A**) Frequency of B220^+^AA4.1^−^CD23^+^ follicular B cells (FO), B220^+^AA4.1^−^CD23^−^IgM^+^ marginal zone B cells (MZ), B220^+^CD138^+^ plasmablasts (PB), and B220^−^CD138^+^ plasma cells (PC) within CD19^+^ B cells (left panel) and frequency of GL7^+^Fas^+^ germinal center–phenotype cells (GC) within CD19^+^IgD^lo^ B cells (right panel) in the spleens of uninfected control mice (WT) and immunocompetent mice born to virus-carrier *Rag1*^−/−^ dams (*Rag1*^−/−^ → WT). Symbols represent individual mice from three separate litters. The *p* value was calculated by the Mann–Whitney rank-sum test. (**B**) Flow cytometric profile (left panel) and frequency (right panel) of CD19^lo^CD23^+^ plasmablasts in B220^+^IgM^+^ B cells from the spleens of the same mice as in (A). The *p* value was calculated by the Mann–Whitney rank-sum test. (**C**) Flow cytometric detection of MHC class II and IgD expression (upper panels) and GL7^+^Fas^+^ germinal center–phenotype cells (lower panels) in the CD19^+^ and CD19^−^ fractions of B220^+^IgM^+^ B cells from immunocompetent mice born to virus-carrier *Rag1*^−/−^ dams (*Rag1*^−/−^ → WT). The plots are representative of four or five mice per group. (**D** and **E**) B220^+^ B cells from CD45.1^+^CD45.2^+^ uninfected control mice and from CD45.1^−^CD45.2^+^ immunocompetent mice born to virus-carrier *Rag1*^−/−^ dams were cotransferred, together with CD4^+^ T cells from EF4.1 *Emv2*^−/−^ mice, into virus-carrier *Rag1*^−/−^ recipients. (D) Flow cytometric separation of B220^+^ B cells in the spleens of these recipients, 15 d after transfer, into CD19^+^IgD^+^ (gate a), CD19^+^IgD^lo^ (gate b), and CD19^−^IgD^−^ (gate c) cells and GL7^+^Fas^+^ germinal center–phenotype cells within gates a–c (upper panels). Donor B cell composition, based on CD45.1 and CD45.2 disparity, in the indicated subpopulations (lower panels). (E) Frequency of B cells from neonatally infected donors (CD45.2^+^ immunocompetent mice born to virus-carrier *Rag1*^−/−^ dams) within the indicated B cell subsets shown in (D). Each symbol is an individual recipient pooled from two independent experiments. The dashed line represents the input frequency.

Ineffective B cell responses in neonatally infected progeny of immunodeficient *Rag1*^−/−^ dams could, in principle, result from relative lack of T cell help rather than intrinsic B cell defects. Detection of RARV2-specific Abs in at least some of the neonatally infected immunocompetent hosts born to immunodeficient *Rag1*^−/−^ dams (Fig. 1C) suggested that not all RARV2-specific B cells were deleted or tolerized. The CD19^+^ B cell compartment in such hosts displayed the typical composition of follicular and marginal zone B cells and plasmablasts, with the exception of plasma cells (B220^−^CD138^+^), which were significantly increased in comparison with uninfected WT mice (Fig. 6A). However, the frequency of germinal center–phenotype (GL7^+^Fas^+^ or GL7^+^CD38^lo^) cells within CD19^+^IgD^lo^ B cells was not significantly different between uninfected and RARV2 neonatally infected WT mice (Fig. 6A), in line with the relative absence of class-switched RARV2-infected cell-binding Abs (Fig. 1C). These findings were indicative of a stunted B cell response in the absence of sufficient T cell help in RARV2 neonatally infected WT hosts. Consistent with this, we also noted the presence of increased frequencies of atypical plasmablasts, which are characterized by downregulation of CD19 expression, in these hosts (Fig. 6B). Indeed, the frequency of CD19^lo^ B2 cells (CD23^+^) in the B220^+^IgM^+^ population was increased significantly in vertically infected WT progeny of immunodeficient *Rag1*^−/−^ dams compared with uninfected WT mice (Fig. 6B). The CD19^lo^ subset in RARV2 vertically infected WT hosts continued to express surface MHC class II and IgD and lacked any germinal center–phenotype (GL7^+^Fas^+^) cells (Fig. 6C), which were suggestive of incomplete differentiation.

To directly evaluate the ability of B cells from RARV2 vertically infected WT hosts to differentiate into plasmablast or germinal center B cells, we compared them with B cells from uninfected WT mice in an adoptive-transfer system. Allotypically marked B220^+^ B cells from RARV2 neonatally infected or uninfected WT mice were cotransferred into virus-carrier *Rag1*^−/−^ hosts (10^7^ of each B cell type per host). These hosts also received CD4^+^ T cells from EF4.1 *Emv2*^−/−^ donors (4 × 10^5^ env-reactive T cells per host), which were not tolerant of *Emv2*-derived RARV2 Ags (28). Fifteen days posttransfer, the B220^+^ population in recipient mice was readily delineated by expression of CD19 and IgD (Fig. 6D). It consisted of CD19^+^IgD^+^ and CD19^+^IgD^lo^ subsets of approximately equal proportions (Fig. 6D). Expectedly, the CD19^+^IgD^lo^ subset also contained all of the differentiated germinal center–phenotype (GL7^+^Fas^+^) cells (Fig. 6D). Importantly, the ratio of the two types of donor B cells remained unchanged in both of these subsets, including the germinal center–phenotype subset (Fig. 6D, 6E), indicating that B cells from RARV2 neonatally infected donors were at least as efficient in giving rise to all of these subpopulations as were B cells from uninfected donors. In addition to the typical B cell subsets, we noted the appearance of a sizable CD19^−^IgD^−^ subset, which was almost exclusively of RARV2 neonatally infected donor origin (Fig. 6D, 6E). Thus, in addition to generating an atypical B220^+^CD19^−^IgD^−^ plasmablast population, B cells from RARV2 vertically infected WT donors competed successfully with B cells from uninfected WT donors in the generation of the typical T cell–dependent germinal center response in virus-carrier hosts.

### T cell help promotes retrovirus-specific B cell responses in neonatally infected hosts

**FIGURE 7.**
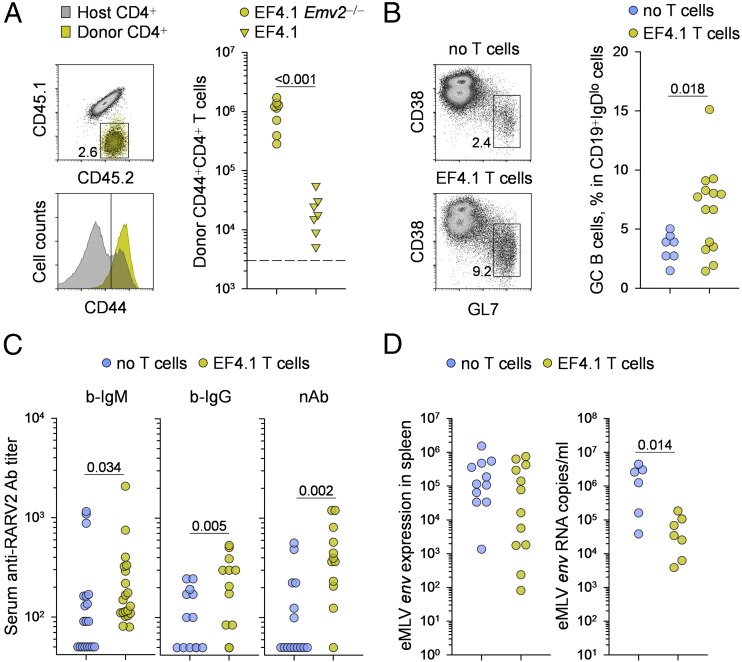
T cell help therapy restores B cell responses in neonatally infected hosts. (**A**) CD45.1^−^CD45.2^+^ CD4^+^ T cells from EF4.1 *Emv2*^−/−^ or EF4.1 donor mice were adoptively transferred into RARV2 neonatally infected CD45.1^+^CD45.2^+^ immunocompetent recipients (born to virus-carrier *Rag1*^−/−^ dams) and recovered 35 d later. Flow cytometric detection and CD44 expression of EF4.1 *Emv2*^−/−^ donor T cells (left panels) and absolute number (right panel) of EF4.1 *Emv2*^−/−^ or EF4.1 donor T cells isolated from the spleens of these recipients. Each symbol is an individual mouse. The dashed line represents recovery from virus-free recipients. The donor-type CD4^+^ T cells recovered from virus-free recipients or from recipients of EF4.1 T cells include CD4^+^ T cells that are not reactive with env. These CD4^+^ T cells vastly outnumber env-reactive T cells in the inoculum. (**B**) Flow cytometric profile (left panels) and frequency (right panel) of GL7^+^CD38^lo^ germinal center–phenotype cells within CD19^+^IgD^lo^ B cells in the spleens of RARV2 neonatally infected immunocompetent mice that did (EF4.1 T cells) or did not (no T cells) receive transfer of EF4.1 *Emv2*^−/−^ CD4^+^ T cells. (**C**) Titers of RARV2-infected cell-binding IgM and IgG Abs and of RARV2-neutralizing Abs in the sera of the same groups of mice as in (B). (**D**) RARV2/*Emv2* eMLV *env* expression, relative to *Hprt* expression, in the spleens (left panel) and copies of RARV2/*Emv2* eMLV *env* RNA in plasma samples (right panel) from the same groups of mice as in (B). In (A)–(D), each symbol is an individual mouse. In (B)–(D), symbols represent individual mice pooled from three independent experiments. All *p* values were calculated by the Mann–Whitney rank-sum test.

Our results supported a model whereby vertical transmission of RARV2 from immunodeficient dams caused central T cell, but not B cell, tolerance in the immunocompetent progeny. According to this hypothesis, the lack of effective B cell responses in these RARV2 neonatally infected mice is due to a deficit in T cell help and not to a B cell defect. If this were true, then the provision of T cell help to RARV2 neonatally infected mice should restore their ability to mount an effective B cell response. To test this hypothesis, we transferred small numbers of allotypically marked CD4^+^ T cells from EF4.1 *Emv2*^−/−^ donors into RARV2 neonatally infected recipients, resulting in engraftment of ∼25,000 RARV2-reactive T cells per recipient spleen. The transferred CD4^+^ T cells activated efficiently (as judged by CD44 upregulation) and maintained a durable response (∼50-fold expansion) until the end of the observation period (day 35) (Fig. 7A). In contrast, CD4^+^ T cells from EF4.1 donors, which were partially tolerant of *Emv2*-derived RARV2 Ags (28), did not expand efficiently under the same conditions (Fig. 7A), and these recipients were not analyzed further. Importantly, EF4.1 *Emv2*^−/−^ CD4^+^ T cell transfer induced a significant expansion of germinal center–phenotype (GL7^+^CD38^lo^) cells within CD19^+^IgD^lo^ B cells in the majority of the recipients (Fig. 7B). Accordingly, serum titers of RARV2-infected cell-binding IgM and IgG Abs, as well as RARV2-neutralizing Abs, were also significantly increased upon CD4^+^ T cell transfer (Fig. 7C).

Last, we examined whether the invigorated RARV2-specific B cell response in neonatally infected recipients of RARV2-specific CD4^+^ T cells translated into an antiviral effect. Expression levels of eMLV env in the spleens of CD4^+^ T cells recipient were not significantly different from RARV2 neonatally infected mice that did not receive CD4^+^ T cells (Fig. 7D). However, vertical transmission of RARV2 results in infection of the majority of spleen cells (Fig. 1B), and B6 mice carry two germline copies of the RARV2 precursor *Emv2* (per diploid genome) in every cell. Thus, it would be an impossible task for the immune system to eliminate cell-associated virus. Therefore, we assessed an effect of adaptive immunity on cell-free virus load. Indeed, plasma viremia was reduced significantly, by an average of 1.3 log_10_, in recipients of RARV2-specific CD4^+^ T cells (Fig. 7D), indicating that T cell help therapy exerts a substantial antiviral effect.

## Discussion

Chronic infection represents a considerable challenge for the host’s immune system because it has to respond to pathogen-derived Ags that persist, often at high levels (43, 44). Such conditions can induce clonal deletion of pathogen-specific lymphocytes, particularly in the developing immune system of the neonate (18, 22, 25). Indeed, immunological tolerance induced by neonatal infection allows for the vertical transmission of viruses through successive generations of otherwise immunocompetent hosts. Using a mouse model for neonatal retroviral infection, we showed that maternal and offspring immunity cooperated to prevent vertical transmission of this virus. Maternal immunodeficiency permitted near-complete central T cell tolerance of the vertically transmitted virus. In contrast, virus-specific B cells were not deleted in the offspring. Maternal immunity protected offspring T cells from central deletion, which allowed the offspring to produce additional T cell–dependent Abs, ultimately purging the virus from subsequent generations.

Immunological tolerance of neonatal infection acquired in the absence of maternal immunity displayed Ag specificity, characteristic of TCR and BCR Ag recognition. Hosts neonatally infected with RARV2 could still effectively defend themselves against challenge with the highly similar FV. Despite their overall similarity, there are immunogenic TCR epitopes that are unique to either virus. For example, a major MHC class I–restricted epitope is present in the leader sequence of glyco-Gag (positions 85–93) of F-MLV (45) but not RARV2 (27). Notably, only 2 of the 17 MHC class II–restricted epitopes that were identified recently in the response of B6 mice to FV (46) are 100% identical to those in RARV2, with the rest exhibiting 0–93% identity. Similarly, F-MLV– and *Emv2*-derived envelope proteins can display distinct Ab epitopes, which is exemplified by the ability of 83A25 mAb to distinguish between F-MLV– and *Emv2*-derived envelopes (40) and the lack of F-MLV cross-reactive Abs in mice vertically infected with RARV2 shown in this study. This degree of Ag specificity argues in favor of TCR and BCR repertoire alterations, rather than extrinsic regulation, as the cause of immunological tolerance to neonatal infection.

Induction of epitope-specific tolerance reported in this article bears striking similarities to findings reported in *H2*^b^-congenic AKR mice (47). Recombination between an eMLV (*Emv11* or *Emv12*) and one or more nonecotropic MLVs in AKR mice generates infectious recombinant MLVs that ultimately cause thymomas (48). AKR mice become immunologically tolerant of the AKR/Gross group of MLVs, even if they carry the responder MHC haplotype *H2*^b^, but they are able to respond to a syngeneic virus-induced tumor expressing Ags of the Friend-Moloney-Rauscher group of MLVs (47, 49). Immunological tolerance of AKR/Gross MLVs in *H2*^b^-congenic AKR mice affects CD4^+^ and CD8^+^ T cells and is due to Fas-mediated peripheral deletion of virus-specific T cell clonotypes (47, 50). Escape from central tolerance of AKR/Gross MLV-specific T cells in *H2*^b^-congenic AKR mice is likely due to the late emergence of infectious recombinant MLVs (48). These earlier studies highlight the potential of MLVs to induce clonal deletion of virus-specific T cells, even after establishment of the peripheral TCR repertoire.

The thymus is responsible for establishing the TCR repertoire available in the periphery to respond to infection. However, the requirement for thymic generation of new pathogen-specific T cells can continue even postinfection, especially of the chronic type (17). Continuous recruitment of newly generated pathogen-specific T cells was suggested to preserve the antiviral CD8^+^ T cell response to persistent infection of mice with polyomavirus or lymphocytic choriomeningitis virus clone 13 (51). These studies underscore the importance of thymic function prior to, as well as during, persistent infection. Consequently, chronic infection of the thymus compromises pathogen-specific T cell immunity in the neonate, as well as the adult, host (23, 24).

Our findings revealed that maternal immunity protected the neonatally infected offspring from thymic deletion of virus-specific T cells. In this model of vertical transmission, the absence of maternal adaptive immunity permitted central T cell tolerance of viral Ags. This effect of maternal immunity is most likely mediated by Abs, which provide a temporary cover that can partially protect the offspring until it can mount its own response. Maternal Abs were shown to promote T cell responses in the offspring in specific settings with important, and often pathogenic, consequences (52–54). Maternal autoantibodies are necessary for the induction of the autoreactive T cell response and ensuing autoimmune disease in NOD mice (54) and a mouse model for autoimmune ovarian disease (52). Similarly, maternal alloantigen-specific Abs were shown to promote an alloantigen-specific T cell response against in utero allogeneic hematopoietic cell transplantation in the offspring (53). The precise mechanisms by which maternal Abs reactive with a given Ag present in the offspring might enhance T cell reactivity against the same Ag remain incompletely understood. However, they are hypothesized to include Ab-mediated enhancement of Ag presentation to T cells, likely through FcγR-mediated Ag–Ab complex uptake by APCs, direct activation of APCs, and Ab-dependent cell-mediated cytotoxicity of Ag^+^ cells (52–54). Alternatively, such maternal Abs might alter the balance between autoantigen-specific effector and regulatory T cells. Our findings support a more direct role for maternal Abs in the thymic generation of the Ag-specific TCR repertoire, rather than a role for the efficiency of peripheral T cell activation. It is possible that the presence of maternal Abs against peripheral autoantigens, alloantigens, or viral Ags in each of these models restricts seeding of the offspring thymus with cells expressing these Ags, thus limiting the degree of central tolerance of these Ags.

In the absence of maternal immunity, neonatal infection resulted in near-complete central tolerance of the antiviral TCR repertoire. Although this work focused of CD4^+^ T cells, whose helper activity is critical for the antiviral B cell response, virus-specific CD8^+^ T cell central or peripheral tolerance is also likely to occur during neonatal infection. Reduction or complete loss of virus-specific CD8^+^ T cells would also facilitate vertical transmission, given the established contribution of CD8^+^ T cells to the control of infectious MLVs derived from endogenous MLV precursors (47). The *env* open-reading frame of endogenous eMLVs, including *Emv2*, encodes an immunodominant CD8^+^ T cell epitope in the transmembrane domain of the envelope protein. The AKR/Gross type of MLVs that arise in AKR mice often escape CD8^+^ T cell recognition of this epitope by mutation or genetic recombination of the *env* open-reading frame (47). The same epitope is also mutated in infectious MLV isolates, such as the *Emv2*-derived helper eMLV of the LP-BM5 retroviral complex, as well as the F-MLV of the FV complex (47). These observations are consistent with strong CD8^+^ T cell–mediated selection of escape mutants during the course of the virological events that lead to thymomas in AKR mice and the emergence of exogenous retroviruses, such as LP-BM5 and FV.

The important contribution of virus-specific CD8^+^ T cells notwithstanding, we focused on immunological tolerance of CD4^+^ T cells, because loss of T cell help alone compromises the magnitude and quality of the antiviral B cell responses, irrespective of any additional effects on the antiviral BCR repertoire. Similarly to the TCR repertoire, central tolerance in the BCR repertoire could also weaken the antiviral B cell response to neonatal infection (2). In principle, developing B cells bearing self-reactive receptors are also subject to central tolerance mechanisms. Indeed, a fraction of self-reactive BCRs are removed from the repertoire, either by receptor editing or apoptosis of the clonotype that harbors them, but another fraction persists in the periphery under a state of functional anergy (2). Studies of B cell tolerance with model Ags yielded conflicting results, and data from chronic infection are lacking (2, 55, 56). Our results argue against clonal deletion or receptor editing in virus-specific B cell precursors in neonatally infected hosts. This premise is supported by the following observations: a population of atypical CD19^lo^ plasmablasts that emerged in neonatally infected hosts expanded upon transfer into infected secondary recipients, indicating virus specificity; many of the neonatally infected hosts mounted readily detectable virus-specific IgM responses, suggesting that at least some virus-specific B cells escaped central tolerance and produced Abs; and restoration of retrovirus-specific T cell help induced a germinal center reaction and the production of virus-neutralizing Abs in the majority of neonatally infected hosts. Thus, our results suggest that failure of the host to mount a B cell response to neonatal retroviral infection is due to insufficient T cell help.

The mechanisms that allow virus-specific B cells to escape central deletional tolerance or receptor editing following neonatal retroviral infection are not entirely clear. Whether chronic Ag recognition by the BCR of immature B cells will induce receptor editing, death, or anergy depends heavily on the nature and dose of the Ag (2, 55, 56). The semicrystalline protein structure of viral particles is considered far more immunogenic that soluble proteins, and virions also provide essential innate signals, rendering them the most potent antigenic form for the B cell response (57). It is possible that, in contrast to other self-antigens, the more immunogenic antigenic stimulation of immature B cells by viral particles prevents their clonal deletion and even promotes their maturation. Notably, B cell responses against endogenous MLVs, which may also form noninfectious virions, develop spontaneously in mice and humans (41, 58, 59). Thus, lack of central B cell tolerance to germline-encoded or vertically transmitted MLVs may represent a general phenomenon. Similarly, the role of CD4^+^ T cells in facilitating central B cell deletion or reversing B cell anergy depends on the quality of BCR signaling and the timing of T cell help. Cognate interaction between a CD4^+^ T cell and an anergic B cell may result in Fas-mediated death of the B cells or in B cell activation (2, 55, 56). Vertically transmitted MLVs centrally delete nearly all virus-specific CD4^+^ T cells that could otherwise facilitate B cell deletion. Elucidation of the precise mechanism by which virus-specific B cells avoid deletional tolerance by endogenous or neonatally acquired MLVs requires further investigation. The findings reported in this study have two main implications. First, they demonstrate that strong T cell and B cell responses to a retrovirus derived from endogenous precursors is possible to induce, even if it is acquired neonatally. Vertically transmitted RARV2 is the product of recombination among the endogenous MLVs *Emv2*, *Xmv43*, and *Xmv8* (27). Although they are all replication defective, they are expressed to a certain degree in normal uninfected B6 mice (60) and induce partial immunological tolerance (28). Nevertheless, deliberate immunization of adult B6 mice can induce T cell responses against *Emv2*-encoded Ags (61), and similar responses to endogenous eMLVs can be induced in *H2*^b^-congenic AKR mice before the onset of peripheral tolerance (47). Moreover, cooperation between maternal and offspring immunity can generate highly neutralizing Abs against RARV2, whose *env* gene is identical to that of the endogenous *Emv2* (27), ultimately breaking the chain of vertical transmission. Thus, strong adaptive-immune responses can be induced against RARV2, despite the fact that it is acquired very early in life and is part of immunological self. T cell and B cell responses against RARV2 or *Emv2*, which are effectively autoimmune in nature, were not associated with any pathological consequences in this model. It would be of interest to investigate whether deliberate induction of anti–endogenous retrovirus responses could be harnessed in the protection of the host against pathological conditions that activate these endogenous retroviruses, such as unrelated infection or cellular transformation (59).

Second, the contrast between efficient central tolerance in T cells and preservation of the potential to mount a B cell response following neonatal retroviral infection reinforces the idea that T cell help may be the limiting factor in the induction of protective Ab immunity in retroviral infection in general. This idea is supported by observations in another model of vertical retroviral transmission (62, 63). Mice of the I/LnJ inbred strain possess a unique genetically determined Ab-mediated ability to resist vertical infection with MLV or mouse mammary tumor virus (62, 63). Although the genetic locus determining this phenotype remains unknown, resistance requires production of IFN-γ by CD4^+^ T cells (62). In our model for retroviral infection of the neonate, immunotherapy of the adult with nontolerized virus-specific CD4^+^ T cells cannot provide sterile immunity because infection affects the majority of the hematopoietic system, and every cell also carries germline copies of the precursor proviruses (27). Nevertheless, CD4^+^ T cell immunotherapy induced significant production of protective Abs, at levels required to block further vertical transmission.

In conclusion, escape of retrovirus-specific B cells from complete central tolerance under conditions of persisting retroviral infection allows T cell help to reignite the antiviral B cell response. Restoration of protective Ab responses by CD4^+^ T cell immunotherapy under the conditions examined advocates the consideration of similar approaches in other chronic infections, particularly when CD4^+^ T cell help is known to be defective, such as in HIV-1 infection.

## Supplementary Material

Data Supplement
