# Supplementary material for: Dichotomy between T Cell and B Cell Tolerance to Neonatal Retroviral Infection Permits T Cell Therapy
Source: J Immunol. 2016 Sep 19;197(9):3628–38. doi: 10.4049/jimmunol.1600734 (PMC5073355; doi:10.4049/jimmunol.1600734)
Supplement: Data Supplement [file JI_1600734.zip › JI_1600734_Supplemental_Figures_1.pdf]

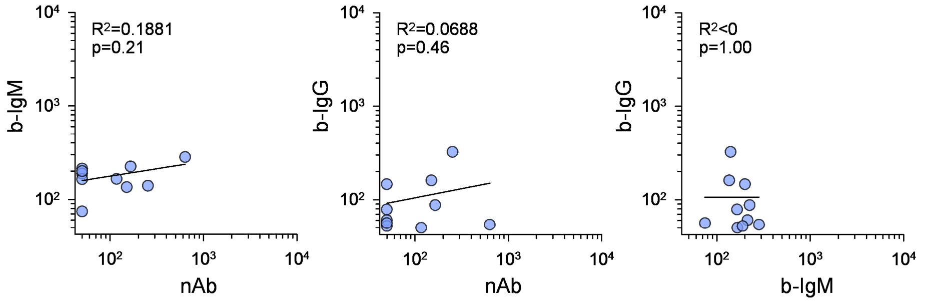

SUPPLEMENTAL FIGURE 1. Correlation between binding antibody classes and neutralizing antibodies in *Rag1*$^{-/-}$ → WT transmission. Scatter plots show the three pair-wise correlations between the titers of RARV2-infected cell-binding IgM antibodies, RARV2-infected cell-binding IgG antibodies and RARV2-neutralizing antibodies in the sera of immunocompetent mice born to virus-carrier *Rag1*$^{-/-}$ dams. Each symbol is an individual mouse.

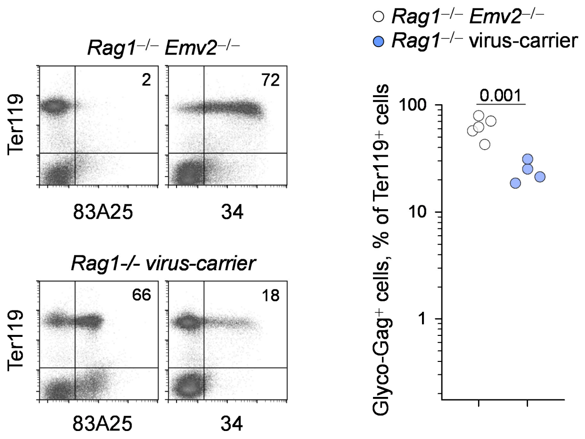

SUPPLEMENTAL FIGURE 2. RARV2 interferes with FV infection. *Left*, Flow cytometric detection of F-MLV and SFFV glyco-Gag with the antibody clone 34, and of endogenous MLV and RARV2 env with the antibody clone 83A25. Plots show total cells and Ter119^{+} erythroid precursors in the spleens of virus-free *Rag1*$^{-/-}$*Emv2*$^{-/-}$ mice, and virus-carrier *Rag1*$^{-/-}$ mice 7 days after FV infection. *Right*, frequency of FV-infected (glyco-Gag^{+}) cells within Ter119^{+} cells in the spleens of the same mice. Each symbol is an individual mouse from a single experiment, representative of two independent experiments. P value was calculated by a Student's t-test.

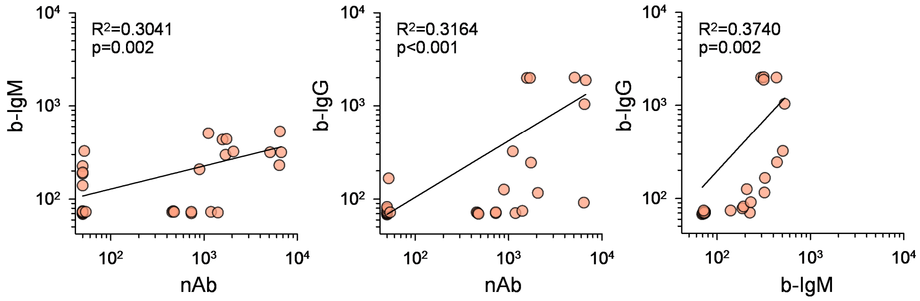

SUPPLEMENTAL FIGURE 3. Correlation between binding antibody classes and neutralizing antibodies in WT → WT transmission. Scatter plots show the three pair-wise correlations between the titers of RARV2-infected cell-binding IgM antibodies, RARV2-infected cell-binding IgG antibodies and RARV2-neutralizing antibodies in the sera of immunocompetent mice born to virus-carrier WT dams. Each symbol is an individual mouse.
